# Discrimination of older peers is associated with workplace age discrimination: moderation by occupational health literacy

**DOI:** 10.1186/s40359-024-02163-0

**Published:** 2024-11-15

**Authors:** Nestor Asiamah, Emelia Sarpong, Usman Yaw Baidoo, Eric Eku, Isaac Aidoo, Etornam Doamekpor, Hafiz T.A. Khan, Emelia Danquah, Cosmos Yarfi, Rita Sarkodie Baffoe, Christiana Afriyie Manu

**Affiliations:** 1 Division of Interdisciplinary Research and Practice, School of Health and Social Care, University of Essex, Colchester, Essex CO4 3SQ United Kingdom; 2 International Public Health Management Programme, University of Europe for Applied Sciences, Reiterweg 26B, Iserlohn, 58636 Germany; 3 Research Faculty, Berlin School of Business and Innovation, 97-99 Karl Marx Strasse, Berlin, 12043 Germany; 4 Africa Center for Epidemiology, Department of Geriatrics and Gerontology, P. O. Box AN 18462, Accra, Ghana; 5 School of Business, Accra Technical University, P. O Box GP 561, Barnes Road, Accra Metro, Accra, Ghana; 6 Department of Business and Social Sciences Education, University of Cape Coast, Private Mail Bag, Cape Coast, Ghana; 7 Department of Secretaryship and Management Studies, Dr Hilla Limann Technical University, Upper West, Wa, Ghana; 8 Department of Building Technology, Accra Technical University, P.O. Box GP 561, Accra, Ghana; 9 Faculty of Business Administration, KAAF University College, P.O. Box Wu 177, Fetteh Kakraba, Central Region Ghana; 10 College of Nursing, Midwifery, and Healthcare, University of West London, Paragon House, Boston Manor Road, Brentford, TW8 9GB United Kingdom; 11 Research Directorate, Koforidua Technical University, Post Office Box KF-981, Koforidua, Eastern Region Ghana; 12 Department of Physiotherapy and Rehabilitation Sciences, University of Health and Allied Sciences, Ho, PMB 31 Ghana

**Keywords:** Ageism, Age discrimination, Correlates, Occupational health, Ageing, Peers

## Abstract

**Background:**

Workplace Age Discrimination Experienced (WADE) can be disorientating and detrimental to well-being. Hence, older employees would like to avoid it, but those who experience it may discriminate against their older peers. WADE may be associated with Age Discrimination of Peers (ADP), and this relationship can be moderated by Occupational Health Literacy (OHL).

**Aim:**

This study aimed to assess the association of WADE with ADP and to ascertain whether this relationship is moderated by OHL.

**Methods:**

A cross-sectional design based on a research-reporting checklist was adopted. Measures against confounding and common methods bias were utlised to avoid or minimise bias. The participants were 1025 middle-aged and older employees (average age = 58 years) who were permanent residents of Accra, Ghana. Hierarchical Linear Regression (HLR) analysis was utilised to analyse the data. Curve estimation was among the methods used to assess assumptions governing HLR analysis.

**Results:**

WADE and OHL were positively associated with ADP, but OHL was negatively associated with WADE in the ultimate models incorporating the covariates. WADE was more positively associated with ADP at moderate and higher OHL, which signified positive moderation of the WADE-ADP relationship by OHL.

**Conclusion:**

Older employees who experience higher age discrimination at work are more likely to discriminate against peers. OHL can be associated with lower WADE but higher ADP. Qualitative studies are needed to understand why OHL may be related to higher ADP.

**Supplementary Information:**

The online version contains supplementary material available at 10.1186/s40359-024-02163-0.

## Introduction

The Decade of Healthy Ageing 2021–2030 is one of the United Nations’ flagship campaigns aimed at fostering healthy ageing by supporting older adults, their families, and the communities where they live [1]. A notable action plan of the initiative is to combat ageism, which the World Health Organization (WHO) defines as the stereotypes, prejudice, and discrimination towards others or oneself based on age [2]. Stereotypes, prejudice, and discrimination respectively refer to the way people think, feel, and act toward others or themselves based on age. Ageism is frowned upon as an antisocial behaviour and has a negative influence on well-being [3–5].

Ageism can take different forms – ageism towards others or oneself and ageism experienced. The type of ageism measured with the Fraboni Scale of Ageism (FSA) [6] is ageism towards others whereas the type measured with the Workplace Age Discrimination Scale (WADS) [7] is ageism experienced. The development of WADS recognises increasing workplace ageism, which is a risk factor for social isolation and job dissatisfaction [3, 4].

Research on workplace ageism is of utmost significance. As the world’s population ages rapidly, the proportion of older people experiencing or perpetrating ageism at work will increase. Full-time employees spend most of their weekday time at work with people [8], implying that these employees are more likely to experience ageism. If so, workplace interventions against ageism would play an important role in meeting the health needs of ageing employees. As the WHO’s definition suggests, there are three domains of ageism: stereotypes, prejudice, and discrimination. Yet, this study focuses on age discrimination since it constitutes harmful actions toward people or oneself [2]. Such harmful actions often emanate from thoughts (stereotypes) and feelings (prejudice), signifying that age discrimination overlaps with the two other domains of ageism. Understandably, many psychometric scales available in the literature [4, 9, 10] measure only age discrimination.

One of the objectives of this study was to examine the association of Workplace Age Discrimination Experienced (WADE) with Age Discrimination toward Peers (ADP). No study has examined this relationship despite having plausible theoretical explanations and the availability of scales measuring WADE and ADP. By assessing the foregoing relationship, we improve stakeholders’ understanding of how ageism may be passed on from person to person. This understanding is needed to develop and roll out programmes to combat ageism. We also draw on the Social Cognitive Theory to conceptualize age discrimination as a potentially contagious behaviour that may require more effort to overcome. It provides insights into how Occupational Health Literacy (OHL) may relate to WADE and ADP in a work context.

OHL refers to the degree to which individuals can access, process, and utilise occupational health information to assess and avoid health risks [11, 12]. Scales measuring OHL include items about the ability to avoid health or safety risks and to “judge negative impact” such as mental health issues associated with ageism [11]. Nevertheless, OHL limits responsible health-seeking action to the workplace and prioritises self-protection at work but not the protection of others outside the organisation. For theoretical reasons explained later, employees who experience less age discrimination at work owing to their high OHL may report a high level of ADP. For such employees, WADE may be more strongly associated with ADP at higher OHL, which signifies a counterintuitive moderation of the WADE-ADP relationship by OHL. This study aimed to further investigate this potential moderation relationship.

This study is the first to examine the relationship between WADE and ADP. It is assumed that OHL protects the individual from health and social risks such as WADE and ADP [11, 12], but there is no evidence on whether it is associated with WADE and ADP. Apart from assessing the association of OHL with WADE and ADP for the first time, this study elucidates implications for designing interventions against ageism. This study provides a model for future research by providing a theoretical perspective on the “contagion of ageism”, which is a situation where a person mimics the ageist behaviour they have experienced from others. Thus, this study demonstrates how likely age discrimination can spread, thereby enabling organizations to identify who may need support (through training) to avoid discrimination against others. It also improves stakeholders’ understanding of age discrimination as a learnable or transferable behaviour. It delineates implications for occupational health policy development and the implementation of campaigns (e.g., the Decade of Healthy Ageing 2021–2030) combating ageism. Finally, this study employs a robust cross-sectional design to guide future research.

### Theoretical framework

We explain the relationship between WADE and ADP with the Social Learning Theory (SLT) and Social Cognitive Theory (SCT). Proposed in 1977 by Albert Bandura, the SLT asserts that behaviours can be acquired by observing and imitating others [13]. Observation is a cognitive process that occurs in a social context where the individual evaluates the social and moral implications of a behaviour and decides whether to perform it. Although observation does not always translate into imitation [13, 14], individuals are more likely to perform a behaviour if they repeatedly observe it. When individuals repeatedly observe a behaviour, they become fond of it through its sustained cognitive processing or view it as an acceptable norm. Both negative and positive behaviours can be deemed acceptable, depending on one’s motivation in an observation. This thought is shared by the SCT.

The SCT was advanced by Albert Bandura as an extension of the SLT [15, 16]. It asserts that observation should include *attention processes*, *cognitive representational processes*, and *motivational process*es. Attention processes refer to a person selectively giving attention to a behaviour depending on its functional value, accessibility, and relevance. Cognitive representational processes refer to the observation of behaviour and its consequences, followed by the conversion of the behaviour into a mental symbol for future reproduction or re-enactment. It is through this process that the individual recalls the behaviour, views it as acceptable or not, and becomes fond of it. The motivational process is about the reproduction of behaviour depending on the responses and consequences received by the individual after re-enacting the behaviour. For example, employees are more likely to repeat a deviant behaviour (e.g., peer abuse) if they were not reprimanded for performing this behaviour in the first instance.

The SCT refers to the process by which people learn through observation or witnessing of behaviour as “modelling”. Although modelling is seen as a desirable process in which good behaviours are learned, it can involve passing on negative traits. This perspective has been used to explain the cognitive and social processes by which parents and peers pass on or acquire negative behaviours. A study in the Netherlands, for example, found that participants’ smoking behaviour was influenced by parents, siblings, and friends in behaviour modelling [17]. Antisocial cognition (which occurs through the observation of negative behaviours) was found to be associated with delinquency among young people. Another study found that experiencing (i.e., observing) childhood abuse, and growing up with domestic violence were associated with intimate partner violence among women [18]. The above studies suggest antisocial behaviours and negative traits can be passed on to young and older adults through “modelling”.

Compared to the SLT, the SCT has a wider conceptual scope, which includes recognizing humans as agents capable of influencing their environment and deciding to perform negative or positive behaviour in social learning. Individuals influence their environment when they choose the right place to model behaviour in self-protection. Through cognition necessitated by social learning, employees can avoid deviant behaviour (e.g., age discrimination of peers) at work (a positive outcome) but manoeuvre to perform this behaviour outside the workplace where they are unlikely to be punished (a negative behaviour). Employees can observe a behaviour (e.g., WADE) at work but choose to mimic it in the form of ADP outside the workplace (i.e., at home or in the neighbourhood). Thus, outcomes of social learning are influenced by the environment, but social learners can also influence their environment by choosing where to perform a learned behaviour. This being so, SCT better explains age discrimination among employees who alternative between two environments (i.e., home/neighbourhood and workplace) where the probabilities of successfully performing a deviant behaviour without being punished are different.

Older adults have rich life experiences that may facilitate good moral judgement. Yet, they are not impervious to acquiring deviant behaviours through negative modelling. This deficiency is attributable to potential “cognitive distortions” associated with observational learning [19]. Cognitive distortions are biased or inaccurate ways of conferring meaning upon everyday experiences [19]. They are also called deficiencies in interpreting social events (including antisocial behaviour). These deficiencies impair moral judgement and compel people to mimic negative traits. If so, older individuals with a desirable moral orientation can confer immoral meanings to experiences (e.g., age discrimination at work) and re-enact them.

Supporting this reasoning is a study in the United States [20] where older adults who showed more negative ageing attitudes reported increased emotional reactivity to daily stressors. A more recent study in Hong Kong found that ageing anxiety is a significant predictor of hostile ageism and ageist microaggressions [21]. The foregoing two studies suggest that moral judgement can be impaired among older adults owing to age-related factors (e.g., ageing anxiety), resulting in undesirable behaviours such as self-directed ageism.

**Fig. 1:**
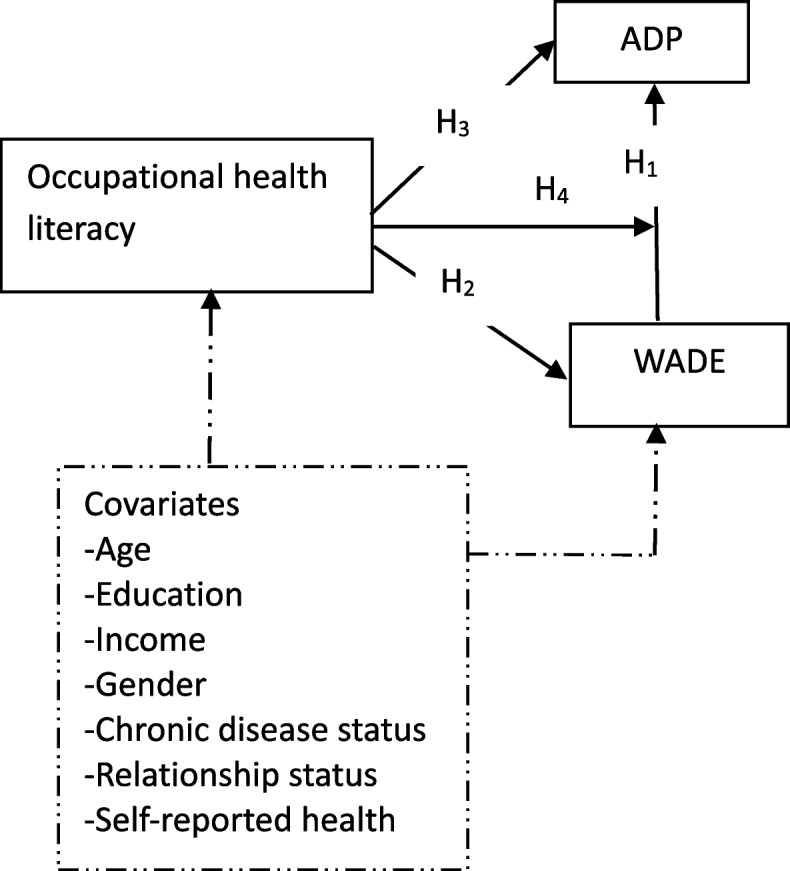
A conceptual model of the association between WADE, ADP, and occupational health literacy. Note: WADE – workplace age discrimination experienced; ADP – age discrimination toward peers, H_1_– the relationship between WADE and ADP; H_2_ – the relationship between occupational health literacy and WADE; H_3_ = the association between occupational health literacy and ADP; H_4_ – moderation of the WADE-ADP relationship by occupational health literacy; broken arrow represents potential confounding effect

Behaviours such as self-directed ageism can be a function of age. A systematic meta-analysis reported two studies that found a positive association between age and self-directed ageism [22], implying that self-directed ageism can be higher in old age. Self-ageism is based on negative perceptions about one’s ageing body that can foster discriminatory attitudes toward older peers. People who can hold ageist views about themselves would easily mimic the discriminatory attitudes of others experienced by them. For such individuals, self-directed ageism would facilitate social learning and re-enactment of age discrimination experienced. As depicted in Fig. 1, therefore, older employees with higher WADE would report higher ADP (hypothesis 1, H1).

Employees are protected from physical, social, and behavioural harms in the workplace through occupational health and safety practices [11, 12]. Through these practices, OHL is fostered in the work environment to enable employees to make health- and safety-seeking decisions. If employees’ actions undermine the values of these practices, they are reprimanded [23]. Employees may lose their incentives, prestigious privileges, or jobs if they perform antisocial behaviour with disregard for occupational health and safety practices. A meta-analysis [23] and the SCT suggest that punishments discourage deviant behaviour in workplaces, especially in contexts where employee supervision is complemented by strict enforcement of organizational laws. As shown in Fig. 1, OHL resulting from occupational health and safety practices would be associated with less WADE (hypotheses 2, H2). Thus, older employees with higher OHL would report lower WADE scores since OHL enables them to use resources and opportunities within the organization to avoid age discrimination.

ADP, unlike WADE, is not limited to the individual’s workplace. Although ADP may occur at work, much of it would be experienced outside the workplace since antisocial behaviour is usually discouraged and reproached in work contexts [23, 24]. The SCT suggests that the propensity for antisocial behaviour increases as one’s observation of this behaviour increases. Repeated modelling of negative traits increases cognitive distortions and impairs moral judgement [19]. It can be inferred from this perspective that deviant behaviours can become compulsive when one gets extremely exposed to them, especially in non-work environments where employees are not subject to closer supervision and enforcement of codes of conduct. Hence, non-work environments (e.g., home and neighbourhood social events) may be viewed as places for practising acquired antisocial behaviours. At the stage where these behaviours are compulsive, employees may exploit such environments to perform antisocial behaviours to make up for opportunities lost at work.

Occupational health and safety practices and OHL typically enable individuals to protect themselves from harm and illness at work [11, 12], not outside the workplace. OHL is a resource for protecting oneself (but not others) with health- and safety-seeking decisions. Although workplace behaviours and values may be applied in other non-work settings, employees are usually not responsible to their employers for how they treat others outside their workplaces. Occupational health and safety practices emphasising self-protection and ethical behaviour at work may lead to a “starvation of cognitive distortions” associated with observing negative traits, which would leave employees with the only chance of utilising these distortions out of proportion in non-work environments. “Starvation of cognitive distortions” refers to a lack of an opportunity to perform behaviours caused by these distortions in the workplace. It is an outcome of organisational codes of conduct discouraging antisocial behaviour at work.

From the preceding viewpoint, antisocial behaviours such as ADP outside the workplace would be high since individuals would imitate them quickly, especially when they become obsessive. ADP outside the workplace would be higher among employees with higher OHL since these individuals may be more conscious of the consequences of ADP at work. Based on the above deductions, we envisage that older employees with higher OHL may report higher ADP (hypothesis 3, H3) and that the association of WADE with ADP may be stronger at higher OHL (hypothesis 4, H4).

As illustrated in Fig. 1, personal factors may confound the above-hypothesised relationships through their influence on WADE and OHL. Studies [25, 26] suggest that ageism or WADE can be associated with personal factors such as age and gender. Older employees may report higher scores of OHL since they have more work experience and have been involved in occupational health and safety practices for a longer period. Human development opportunities available to men and women in African workplaces are unequal [25], so OHL may be related to gender. Women and older adults are more likely to report WADE [26, 27]. Employees with one or more chronic diseases (i.e., a chronic disease status) are more vulnerable and are, therefore, likely to experience higher workplace age discrimination. Similarly, employees with less income are more vulnerable to workplace age discrimination. It was, thus, necessary to consider the covariates in Fig. 1 as potential confounders of the hypothesized relationships. 

## Methods

### Design

A cross-sectional design based on the Strengthening the Reporting of Observational Studies in Epidemiology (STROBE) checklist was adopted. This design included procedures against common methods bias and confounding.

### Participants, sample size, and recruitment

The participants were adult employees of service and manufacturing organizations in Accra, Ghana. The following inclusion criteria were used to select the participants: (i) being aged 50 years or older; (ii) being an employee at the time of data collection, and (iii) willingness and availability to participate in the study. In the selection process, potential participants were interviewed at community centres, malls, and supermarkets by research assistants to identify those who met the above inclusion criteria. A total of 1455 individuals met the inclusion criteria. The minimum sample was calculated with the Daniel Soper online sample size calculation tool for HLR. This tool has been reliably used by previous researchers [28, 29]. The sample size calculated based on recommended parameters (effect size – 0.15; α – 5%, power – 80%, and maximum number of predictors – 11) was 122. We aimed to collect data on all 1455 eligible participants to maximise the power of our tests.

### Variables and measurement

The dependent variable, ADP, was measured with the age discrimination component of the FSA adopted in whole with four descriptive anchors (i.e., strongly disagree – 1, disagree – 2, agree – 3, and strongly agree – 4) from a previous study [6]. This sub-scale constitutes 7 items and measures the extent to which an individual discriminated against others based on age over the past week. Some of its items are “Elderly people should find friends in their age group” and “Elderly people don’t really need to use our community sports or recreational facilities”. The scale’s seventh item was negative and was, therefore, reverse-coded. The scale had satisfactory internal consistency (i.e., Cronbach’s α = 0.82). Scores from this scale range from 7 to 28, with larger scores indicating higher age discrimination.

WADE, the independent variable, was measured with the WADS, a 9-item tool adopted in whole from the literature [7]. It accompanied five descriptive anchors (i.e., 1 – never, 2 – rarely, 3 – sometimes, 4 – often, and 5 – very often) and measured how often employees experienced age discrimination at work over the past week. Some of its items are “I have been passed over for a work role/task due to my age” and “I receive less social support due to my age”. Scores on the scale range from 9 to 45, where larger scores represent higher WADE. Its Cronbach’s α was 0.92.

OHL was measured with a 12-item standard scale adopted in whole with its four descriptive anchors (i.e., 1 – strongly disagree, 2 – disagree, 3 – agree, and 4 – strongly agree) from the literature [11]. Some of its items are “I find safety and health information”, and “I speak about health risks”. It yielded a satisfactory internal consistency (i.e., Cronbach’s α = 0.72). Scores on this scale range from 12 to 48, with larger scores representing higher OHL. Appendix A shows all items of the scales used to measure ADP, WADE, and OHL.

We measured demographic and personal characteristics (i.e., age, education, income, gender, marital status, self-reported health, and chronic disease status) as potential covariates. Age, education, and income were measured as discrete variables. Age was measured as the individual’s chronological age whereas education was measured as one’s years of schooling. Income was the individual’s net monthly earnings in Ghana cedis (₵). Gender (i.e., men – 1, and women – 2), chronic disease status (i.e., none – 1, and one or more – 2), marital status (i.e., not married – 1, and married – 2), and self-reported health (i.e., poor – 1, and good – 2) were measured as categorical variables. All categorical variables were coded into dummy-type variables for statistical analyses. We measured chronic disease status by asking participants to report the number of chronic illnesses they had. We then put the responses into two categories (i.e., those who did not report any chronic disease and those who reported one or more chronic diseases).

### Instrumentation

A self-reported questionnaire was utilised to collect data. The first, second, and third blocks of the questionnaire presented measures of WADE, ADP, and OHL respectively. Preambles guiding participants to understand the context of the scales and answer their respective questions were part of these blocks. The fourth section presented the demographic attributes of the participants. A general introductory section presenting the aim of the study, general survey completion guidelines, and information about informed consent and ethics preceded the above blocks.

At the research design stage, we followed procedures in the literature [30, 31] to avoid or minimise common method bias. Firstly, participants were guided to accurately complete the questionnaire and scales. We ensured participants understood the contents of the questionnaire before completing it. By putting questionnaire information in blocks, we enabled participants to answer questions in the unique context of each scale. Finally, we followed Harman’s one-factor statistical method to assess common methods bias. As a part of this procedure, Exploratory Factor Analysis (through the maximum likelihood method) was used to explore the factor structures of the three scales. As recommended in the literature [30], all scales produced at least two factors (WADE – 2 factors; ADP – 3 factors, and OHL – 3 factors) and the variance contributed by each factor was less than 40%. This outcome confirmed that common methods bias was avoided or minimised.

### Data collection

Research assistants administered questionnaires in person at the centres where the participants were recruited. Participants who could not complete and return their questionnaires instantly were allowed to do so in two weeks. Such participants returned completed questionnaires in labelled envelopes provided by the researchers. Data were collected over four weeks between July and August 2023. Four hundred and thirty questionnaires were not returned or were partially completed. Hence, 1025 questionnaires were analysed.

### Statistical analysis method

**Fig. 2:**
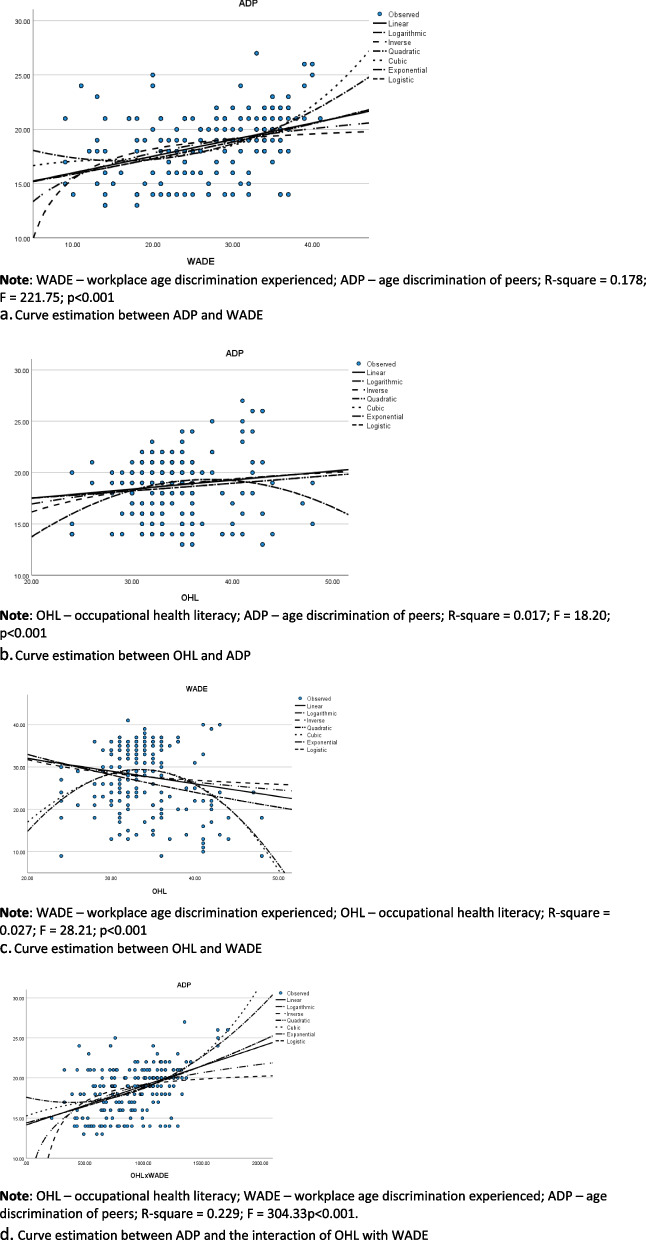
**a** Curve estimation between ADP and WADE. **b** Curve estimation between OHL and ADP. **c** Curve estimation between OHL and WADE. **d** Curve estimation between ADP and the interaction of OHL with WADE

Data were analysed with SPSS 28 (IBM Inc., New York, USA) at two stages. In the first stage, the data were summarised with descriptive statistics, missing data were analysed, and statistical assumptions governing the use of HLR were assessed. Based on guidelines in the literature [32], education or “years of schooling” was removed through listwise deletion of missing data. Relevant assumptions (i.e., linearity of the relationships, multicollinearity, independence of errors, and normal distribution of the data) governing HLR analysis were assessed with procedures adopted from previous research [8]. Through the curve estimation procedure, the linearity of the relationships was confirmed. Figure 2 depicts the linearity of all the relationships tested, and Appendix B shows other assumptions considered and methods for examining them.

In the second stage, Pearson’s zero-order correlation coefficients between relevant variables were computed. Subsequently, six HLR models were fitted to assess the hypothesized relationships, and the first three of them were baseline (non-adjusted) models without the covariates. The first baseline model examined the association of WADE and OHL with ADP whereas the second one assessed the association of only OHL with WADE. The third baseline model tested the association of WADE, OHL, and the interaction between them (i.e., OLHxWADE) with ADP. Following standard practice [33], we created the interaction term by mean-centring the moderator (i.e., OHL) and using the compute function in SPSS to multiply the mean-centred variable with WADE. Models 4, 5, and 6 were the adjusted versions (i.e., models including the covariates) of models 1, 2, and 3 respectively. In a sensitivity analysis, we ascertained the stability of standardised regression weights by comparing the weights between adjusted and non-adjusted models. A chart depicting the association of WADE with ADP at different levels of OHL was plotted to illustrate the interaction of OHL and WADE on ADP. The statistical significance of the results was detected at a minimum of *p* < 0.05.

## Results

In Table 1 are the summary statistics of all variables. About 45% (*n* = 460) of the participants were men whereas the average age of the participants was about 58 years (Mean = 58.23; SD = 6.89). The average WADE, ADP, and OHL were respectively about 28 (Mean = 27.87; SD = 7.96), 19 (Mean = 18.73; SD = 2.88), and 34 (Mean = 33.87; SD = 4.35). Table 2 shows Pearson’s (zero-order) correlation coefficients among the variables. There was a moderate positive correlation between WADE and ADP (*r* = 0.46, *p* < 0.001, two-tailed), suggesting that older employees who experienced higher age discrimination at work reported higher age discrimination of older peers. OHL was negatively correlated with WADE (*r* = -0.164, *p* < 0.001, two-tailed) but positively correlated with ADP (*r* = 0.132, *p* < 0.001, two-tailed).

**Table 1:** Summary statistics on variables included in the analysis (*n* = 1025)

Variable	M	SD	Range	*n* (%)
WADE	27.87	7.96	9–41	
OHL	33.87	4.35	24–48	
ADP	18.73	2.88	13–27	
Age (yrs)	58.23	6.89	50–81	
Education (yrs)	22.12	4.32	15–28	
Income (₵)	1704.33	1992.76	450–2500	
Gender				
Men				460(45%)
Women				565(55%)
Chronic disease status				
None				240(23%)
One or more				785(77%)
Relationship status				
No				345(34%)
Yes				680(66%)
Self-reported health				
Poor				260(25%)
Good				765(75%)

*WADE *Workplace age discrimination experienced, *OHL *Occupational health literacy, *ADP *Age discrimination of peers, *M *Mean, *SD *Standard deviation, *n *Frequency

**Table 2:** Zero-order correlation matrix of variables (*n* = 1025)

Variable	1	2	3	4	5	6	7	8	9
1. WADE	1	− .164**	.422**	.170**	− .230**	.063*	− .116**	− .297**	-0.032
2. OHL		1	.132**	0.01	.076*	− .149**	.138**	0.035	-0.041
3. ADP			1	.171**	− .209**	-.052	-0.031	− .167**	− .075*
4. Gender				1	− .084*	0.011	.146**	− .075*	0.019
5. Income (₵)					1	.081*	.081*	.086*	− .133**
6. CDS						1	− .199**	− .269**	.219**
7. RS							1	.297**	− 0.312**
8. SRH								1	− .090**
9. Age (yrs)									1

*WADE *Workplace age discrimination experienced, *OHL *Occupational health literacy, *ADP *Age discrimination of peers, *CDS *Chronic disease status, *RS *Relationship status, *SRH *Self-reported health

**p < 0.001

*p < 0.05

**Fig. 3:**
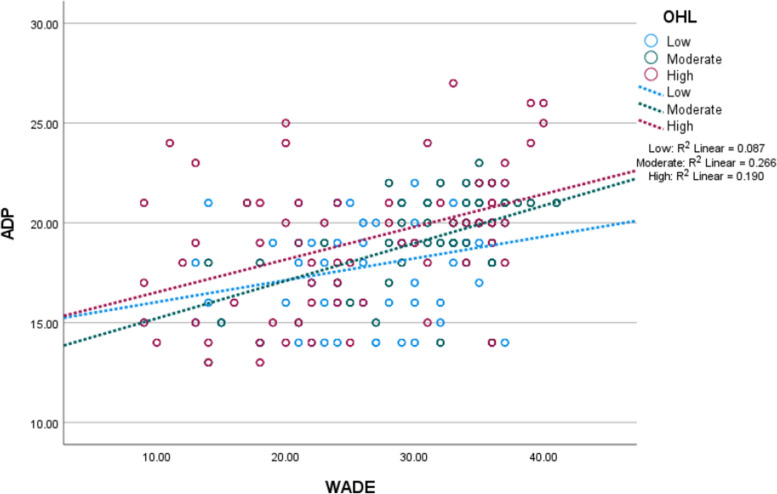
Association of workplace age discrimination experienced and age discrimination of peers at different levels of occupational health literacy (*n* = 1025, low = 341, moderate = 342, high = 342). Note: WADE – workplace age discrimination experienced; ADP – age discrimination of peers

The results of standardised regression coefficients (β) are used here for comparison among selected covariates. Table 3 shows HLR analysis results based on the above correlations. Considering model 4 of Table 3, WADE (β = 0.46; t = 13.12; *p* < 0.001) and OHL (β = 0.15; t = 4.63; *p* < 0.001) were positively associated with ADP. This result confirmed that older employees who experienced higher workplace age discrimination and had higher OHL reported larger scores of older peer discrimination. However, in model 6 OHL was found to be negatively associated with WADE (β = -0.25; t = -7.35; *p* < 0.001), which suggests older employees with higher OHL experienced lower workplace age discrimination. The interaction between OHL and WADE was positively associated with ADP (β = 0.45; t = 13.88; *p* < 0.001). The effect size corresponding to the interaction term was 2% lower than the effect size between WADE and ADP. In Fig. 3, WADE more strongly predicts ADP at moderate and higher OHL, compared to low OHL. Some effect sizes differ between the adjusted and non-adjustment models. For example, the effect sizes of OHL in models 3 and 6 were different.

**Table 3:** Association of age discrimination of peers with workplace age discrimination experienced and occupational health literacy (*n* = 1025)

Model	Variable	Regression Coefficients			95% CI (of B)	Model fit summary			
		B	SE	β		*R* ^2^	Adjusted *R*^2^	Durbin-Watson	F-statistic
Baseline (unadjusted) models									
1	(Constant)	9.50	0.73	(12.94)***	± 2.88	0.22	0.22	1.79	143.96***
	WADE	0.17	0.01	0.46(16.28)***	± 0.04				
	OHL	0.14	0.02	0.21(7.39)***	± 0.07				
2	(Constant)	38.03	1.93	(19.72)***	± 7.57	0.03	0.03	---	28.21***
	OHL	-0.30	0.06	-0.16(-5.31)***	± 0.22				
3	(Constant)	17.59	1.96	(8.98)***	± 7.69	0.24	0.23	1.72	104.24***
	OHL	-0.09	0.06	-0.14(-1.68)	± 0.21				
	WADE	-0.18	0.08	-0.49(-2.28)*	± 0.30				
	OHLxWADE	0.01	0.00	0.66(4.45)***	± 0.01				
Ultimate (adjusted) models									
4	(Constant)	7.31	1.61	(4.53)***	± 6.33	0.28	0.27	1.98	36.07***
	WADE	0.15	0.01	0.46(13.12)***	± 0.05				
	OHL	0.11	0.02	0.15(4.63)***	± 0.09				
	Gender	0.80	0.17	0.15(4.62)***	± 0.68				
	Income (₵)	0.00	0.00	-0.10(-2.92)**	± 0.00				
	CDS	0.03	0.22	0.00(0.13)	± 0.84				
	RS	-0.51	0.21	-0.09(-2.40)**	± 0.83				
	SRH	0.61	0.21	0.11(2.91)**	± 0.83				
	Age (yrs)	0.05	0.02	0.08(2.45)*	± 0.08				
5	(Constant)	46.33	4.74	(9.78)***	± 18.60	0.19	0.19	1.78	25.35***
	OHL	-0.51	0.07	-0.25(-7.35)***	± 0.27				
	Gender	2.02	0.54	0.13(3.76)***	± 2.11				
	Income (₵)	0.00	0.00	-0.17(-5.01)***	± 0.00				
	CDS	-0.44	0.67	-0.02(-0.65)	± 2.64				
	RS	-1.15	0.66	-0.07(-1.75)	± 2.58				
	SRH	-2.92	0.65	-0.17(-4.51)***	± 2.54				
	Age (yrs)	0.10	0.06	0.06(1.66)	± 0.24				
6	(Constant)	22.29	3.10	(7.20)***	± 12.16	0.31	0.30	1.94	36.90***
	OHL	-0.30	0.08	-0.43(-3.96)***	± 0.30				
	WADE	-0.40	0.10	-0.56(-4.02)***	± 0.39				
	OHLxWADE	0.004	0.00	0.45(13.88)***	± 0.01				
	Gender	0.76	0.17	0.14(4.45)***	± 0.67				
	Income (₵)	0.00	0.00	-0.10(-3.12)**	± 0.00				
	CDS	-0.18	0.21	-0.03(-0.83)	± 0.84				
	RS	-0.49	0.21	-0.08(-2.38)*	± 0.81				
	SRH	0.46	0.21	0.08(2.21)*	± 0.82				
	Age (yrs)	0.06	0.02	0.10(2.91)*	± 0.08				

The dependent variable is ADP in all models except model 5; the dependent variable in model 5 is WADE; the “tolerance” value for each predictor is ≥0.6, except in models 3 and 6

*B *Unstandardised effect sizes, *β *Standardised effect sizes, *SE *Standard error (of B), *CI *Confidence interval, *WADE *Workplace age discrimination experienced, *CDS *Chronic disease status, *RS *Relationship status, *CDS *Chronic disease status

***p<0.001

**p<0.01

*p<0.05

## Discussion

This study aimed to assess the association of WADE with ADP and to ascertain whether this relationship is moderated by OHL. Covariates were adjusted for testing the hypotheses with HLR after relevant assumptions (e.g., linearity of the relationships) were assessed.

This study found a positive association of WADE with ADP, which means participants who reported higher WADE were more likely to discriminate against peers. This outcome reflects the likelihood of older employees mimicking the age discrimination they experience and supports the SCT’s view that people can mimic behaviours through observation of others in a social context [13, 14]. The SLT similarly posits that reenactment of behaviour can be the outcome of learning other people’s behaviours and deciding whether the mimicked behaviour is worth performing.

In the absence of policies that require the retribution of organizational outlaws, individuals are more likely to practice negative traits observed and learned [23]. As such, employees with higher WADE possibly learned to discriminate against others at work but only practiced ADP outside the organization where they are not responsible to their employers for how they treat others. Alternatively, employees who experienced higher workplace age discrimination reported higher ADP because authorities in their organizations overlooked deviant behaviours and failed to reprimand their perpetrators. Although no study has examined the association of WADE with ADP, the above result is analogous to evidence from studies that investigated the association between deviant behaviours and social learning [17, 18]. These studies are comparable to the current study because their effects and associations were based on negative modelling or social learning of behaviours in social contexts transferrable to workplaces and older employees.

Occupational health and safety practices, on which OHL depends [11, 34], are generally designed to enable employees to remain safe and healthy. To reiterate, knowledge and resources from these practices that are a part of OHL scales enable employees to protect themselves from behavioural and health-related harms. Items such as I “judge negative impact” and “understand information” on the OHL scale represent the ability to identify and avoid age discrimination. Collectively, items of the scale signify one’s preparedness against workplace anti-social behaviours. In an organization where these behaviours are frowned upon and reproached by employees and authorities, individuals are unlikely to discriminate against their peers based on age. This reasoning is supported by the negative association between OHL and WADE, an outcome implying that individuals with higher OHL experienced less workplace age discrimination.

A seemingly counterintuitive result, on the other hand, is the positive moderation of the WADE-ADP relationship by OHL. Thus, WADE more strongly predicts ADP positively at higher OHL. This result is consistent with our argument regarding the likelihood of people excessively mimicking a behaviour outside the workplace when organisational rules and practices prevent them from performing acquired but compulsive behaviours. The SCT recognises this behaviour as an outcome of people’s capability (e.g., self-regulation) to influence their environment in a social learning process. With self-regulation, employees can choose where and when to perform a behaviour in self-defence.

Social learning is usually a continuous cognitive process that can continuously increase the propensity for performing a behaviour [16, 19, 35]. Consequently, the urge to perform a negative behaviour can reach a level where the behaviour becomes compulsive. At this stage, employees with the above-mentioned capability may exploit non-work environments to perform antisocial behaviour to make up for opportunities lost at work. This viewpoint better applies to employees with higher OHL since they would be more conscious of the consequences of ADP in their workplaces. The re-enactment of a negative trait outside an ethical organisation is more likely among employees who, owing to their higher OHL, cannot mimic this behaviour at work but cannot withstand the impulse to practice it somewhere else.

### Implications for practice

The positive association between WADE and ADP suggests that employees are not only vulnerable to external discrimination (i.e., age discrimination by others) but also have the propensity to acquire it through learning. It also signifies the contagion of age discrimination since individuals who have experienced and acquired it are more likely to display it when dealing with others. It can, thus, be inferred that age discrimination can spread in workplaces where a culture of zero tolerance for it is not enforced. Workplaces without policies forbidding age discrimination or chastising individuals who perform antisocial behaviour may experience a spread of ageism. Ageism from one employee can spread across the social fabric of the organization, which can encourage turnover, increase mental health risks (e.g., depression, anxiety, and social isolation), and weaken employee engagement.

Organisations do not only need anti-ageism policies but also need to roll out programmes to identify those who are acquiring age discrimination as a negative trait. This step is necessary for identifying the social networks or individuals through which age discrimination can spread at work. Employees may acquire age discrimination from outside the workplace and practise it within the organization. This possibility should motivate employers to redesign their occupational health and safety practices toward facilitating positive behaviours beyond the workplace. Redesigned occupational health and safety practices should enhance the scope of OHL, enabling employees to avoid deviant behaviours (not only physical harm) both at work and outside the workplace. A way to enhance the scope of OHL is implementing educational and training programmes that enable employees to recognise ageism as an acquirable trait. Such programmes should enable employees to protect themselves and others from physical and behavioural harm. Practices should not focus on self-protection within the organization.

The positive WADE-ADP relationship unfolds the likelihood of WADE being associated with higher self-age discrimination. In other words, self-age discrimination can be the consequence of experiencing age discrimination from others. There is a need for this possibility to be investigated by filling some research gaps in the literature. A notable gap is the absence of a scale measuring self-age discrimination and research assessing the relationship between age discrimination experienced and age discrimination towards others. The foregoing deductions and recommendations may be overstated for results from a cross-sectional design but would hold more meaning if experimental designs were utilised to confirm the effects reached in this study.

The SLT and SCT agree that social learning involves observing behaviour and possibly mimicking the behaviour. Whether one will mimic the behaviour depends on several factors, including the consequences of modelling the behaviour. This shared import of the two theories would help employers see the possibility of victims of ageism more strongly discriminating against their colleagues based on age. This view reveals the need for organizations to adopt a policy and culture of zero-tolerance for age discrimination. Such a policy must emphasize open punishment of employees who discriminate against their colleagues based on age, given that punishment is the only way to discourage the modelling of negative behaviours. The SCT helps organizations to appreciate the possibility of employees modelling negative behaviours learned at work outside the workplace, or vice versa. If so, punishing culprits of ageism would discourage the negative modelling of antisocial behaviours at work. Punishment must, nonetheless, be ethical and consistent with the law.

Organizations can avoid age discrimination and its spread through age-blinded staff recruitment, intergenerational workplace activities, the provision of equal opportunities for development, and unbiased representation of older adults in senior management roles. An age-blinded recruitment is a process in which selection decisions are not based on the job applicant’s age. Age discrimination is avoided in this process because it does not allow the selection panel to make decisions based on applicants’ ages. Intergenerational workplace activities are recreational and operational activities in which participants from all age groups in the organization are equally represented. These activities can foster social cohesion between age groups and signal zero tolerance for ageism in the organization. Policy-driven training for enabling employees to avoid age discrimination and report culprits of ageism should be regularly rolled out. All age groups should be equally represented in training activities. Finally, organizations’ reward and recognition schemes should not be based on how young employees are.

### Limitations and strengths

Non-probability sampling was utilised in this study, and the participants were from only Accra. Although the sample size used is relatively large, it may not be representative of older employees in Accra. Hence, future studies utilising samples representative of Accra may yield different outcomes, and our results may not be generalised to other populations. All measures employed were self-reported and susceptible to response bias, which may have led to over- or under-estimated WADE, OHL, and ADP. The cross-sectional design employed meant that causation between the variables was not possible, implying that regression weights reported in this study should be viewed as bidirectional associations. Other covariates such as “previous experience of discrimination,” ethnicity, and job rank can confound the association assessed. Some ethnic groups, for instance, may experience higher discrimination and, therefore, more strongly discriminate against others. This being so, future research should include potential covariates not considered in this study.

Ghana’s retirement age of 60 years [36] is low, compared to the retirement ages of western countries such as the United Kingdom and Germany. Compared with these Western countries, Ghana is a relatively young country with a smaller proportion of older employees [36]. This demographic profile of Ghana implies that the levels of WADE and ADP reported in this study are probably different from levels in older employees in Western countries. This being so, our results may not be supported in countries where the retirement age is over 60 years. Future replication of this study in Western countries or contexts where the retirement age is above 60 years is necessary.

This study has several strengths. Firstly, it is the first to examine the relationship between WADE, ADP, and OHL. Secondly, it employs a robust cross-sectional design based on the Strengthening the Reporting of Observational Studies in Epidemiology (STROBE) checklist. Appendix C shows the STROBE checklist on which the study was based. It also assesses relevant assumptions (i.e., linearity of relationships) that previous studies on the topic failed to consider, thereby providing a model for better designing cross-sectional studies. Steps were taken to avoid common methods bias associated with the use of self-reported psychometric measures. This study, thus, serves as a model for designing future cross-sectional studies. By controlling for potential covariates by comparing the adjusted and non-adjusted models, we avoided or minimised confounding bias.

## Conclusion

WADE and OHL were positively associated with ADP, which suggests that older employees who experienced higher workplace discrimination and had higher OHL reported higher discrimination of their peers. Despite being positively associated with ADP, OHL was negatively associated with WADE. WADE was more strongly associated with ADP at moderate and higher OHL, which signified a positive moderation of the WADE-ADP relationship by OHL. We conclude that older employees who experience higher age discrimination at work are more likely to discriminate against their older peers. Organizational practices for improving OHL can buffer WADE but may also be associated with higher ADP. Qualitative studies exploring how current occupational health and safety practices can be designed to buffer both WADE and ADP are needed. We call for similar studies examining experiences and opinions about why OHL may be associated with higher ADP. Increasing OHL among employees would not be sufficient for discouraging ADP outside the organization, and efforts to combat ageism should include more stringent law enforcement against perpetrators of ageism.

## Supplementary Information

Supplementary Material 1.

Supplementary Material 2.

Supplementary Material 3.

## Data Availability

The data are attached as "data file".
