# Supplementary material for: Discrimination of older peers is associated with workplace age discrimination: moderation by occupational health literacy
Source: BMC Psychol. 2024 Nov 15;12:662. doi: 10.1186/s40359-024-02163-0 (PMC11566144; doi:10.1186/s40359-024-02163-0)
Supplement: Supplementary file 1 — Supplementary Material 1. [file 40359_2024_2163_MOESM1_ESM.doc]

**Appendix A**

**Appendix A1 – Measures of age discrimination toward others (ADP)**

**Part 2: Ageist (Self) Discrimination**

On a scale of 1 to 4, **where 1 – strongly disagree, 2 – disagree, 3 – agree, and 4 – strongly agree**, indicate what your views are about older employees at your workplace.

| SN | Item/Statement | 1 | 2 | 3 | 4 |
| --- | --- | --- | --- | --- | --- |
| 1 | Elderly people don’t really need to use our community sports or recreational facilities |  |  |  |  |
| 2 | I don’t like it when elderly people try to make conversation with me |  |  |  |  |
| 3 | Feeling depressed when around elderly people is probably a common feeling |  |  |  |  |
| 4 | Elderly people should find friends in their own age group |  |  |  |  |
| 5 | Complex and interesting conversation cannot be expected from most elderly people |  |  |  |  |
| 6 | It’s best that elderly people live where they won’t bother anyone |  |  |  |  |
| 7 | Elderly people should be encouraged to speak out politically (R) |  |  |  |  |

**Appendix A2. Measures of workplace age discrimination experienced (WADE)**

On a scale of 1 to 5, **where 1 – never, 2 – rarely, 3 – sometimes, 4 – often, and 5 – very often**, indicate how often you experience age discrimination at your workplace.

| SN | Statement/item | 5-item scale | | | | |
| --- | --- | --- | --- | --- | --- | --- |
|  |  | 1 | 2 | 3 | 4 | 5 |
| 1 | I have been passed over for a work role/task due to my age |  |  |  |  |  |
| 2 | My contributions are not valued as much due to my age |  |  |  |  |  |
| 3 | I have been given fewer opportunities to express my ideas due to my age |  |  |  |  |  |
| 4 | I have unfairly been evaluated less favourably due to my age |  |  |  |  |  |
| 5 | I receive less social support due to my age |  |  |  |  |  |
| 6 | I have been treated as though I am less capable due to my age |  |  |  |  |  |
| 7 | I have been treated with less respect due to my age |  |  |  |  |  |
| 8 | Someone has delayed or ignored my requests due to my age |  |  |  |  |  |
| 9 | Someone has blamed me for failures or problems due to my age |  |  |  |  |  |

**Appendix A3. Items for measuring occupational health literacy**

On a scale of 1 to 4, **where 1 – strongly disagree, 2 – disagree, 3 – agree, and 4 – strongly agree**, indicate how well you can perform the following tasks at your current workplace.

| SN | Potential/ability | 1 | 2 | 3 | 4 |
| --- | --- | --- | --- | --- | --- |
| 1 | Find safety and health information |  |  |  |  |
| 2 | Judge negative impact |  |  |  |  |
| 3 | Understand information |  |  |  |  |
| 4 | Implement proactive solutions |  |  |  |  |
| 5 | Change working conditions for health |  |  |  |  |
| 6 | Speak about health risks |  |  |  |  |
| 7 | Evaluate health promotion services |  |  |  |  |
| 8 | Find information about health risks |  |  |  |  |
| 9 | Informed about rules of conduct |  |  |  |  |
| 10 | Assume responsibility for health |  |  |  |  |
| 11 | Discuss health with others |  |  |  |  |
| 12 | Participate in active health promotion |  |  |  |  |
