# Supplementary material for: Discrimination of older peers is associated with workplace age discrimination: moderation by occupational health literacy
Source: BMC Psychol. 2024 Nov 15;12:662. doi: 10.1186/s40359-024-02163-0 (PMC11566144; doi:10.1186/s40359-024-02163-0)
Supplement: Supplementary file 2 — Supplementary Material 2. [file 40359_2024_2163_MOESM2_ESM.doc]

Appendix B. Steps to taken to test five necessary assumptions for HLR analysis

| # | Assumption | Step | Result | Decision |
| --- | --- | --- | --- | --- |
| 1 | Normality of the data associated with the dependent variable | We computed the Mahalanobis values through the HLR in which ADP was the dependent variable | The significance values associated with the Mahalanobis values met the condition p<0.001; thus normality was confirmed | We proceeded with the parametric analysis since the condition was met. |
| 2 | Linearity | We performed curve estimation for each of the hypothesised relationships shown in Figure 1 | Linearity was confirmed at p<0.001. | Assumption or condition met for HLR analysis |
| 3 | Independence of errors | Durbin Watson statistics were generated for all the HLR models fitted. | Durbin-Watson statistic was approximately 2 for each multiple regression model as recommended | The assumption was met for HLR analysis |
| 4 | Multi-collinearity | Tolerance values were computed through the above HLR analysis | The tolerance values are >0.2 as recommended | The assumption was met for HLR analyses |
| 5 | Homogeneity of variances | We plotted standardized residuals against standardized predicted values of the dependent variable in all HLR models | The graphs produced a satisfactory pattern as recommended | The assumption was met for HLR analyses |

**Note**: ADP – age discrimination of peers; HLR – hierarchical linear regression
